# Photothermal Porous Material with Gradient Hydrophobicity for Fast and Highly Selective Oil/Water Separation and Crude Oil Recovery

**DOI:** 10.3390/biomimetics10090585

**Published:** 2025-09-03

**Authors:** Tianwen Wang, Song Song, Shiwen Bao, Yanfeng Gong, Yujue Wang, Chuncai Wang, Wenshao Ma, Nuo Liu, Kunyan Sui, Jun Gao, Xueli Liu

**Affiliations:** 1 Key Laboratory of Marine Bio-Based Fibers of Shandong Province, Key Laboratory of Shandong Provincial Universities for Advanced Fibers and Composites, College of Materials Science and Engineering, Qingdao University, Qingdao 266071, China; 19860912252@163.com (T.W.);; 2 The Chinese Academy of Sciences Includes the Qingdao Institute of Bioenergy and Bioprocess Technology, Qingdao 266101, China; 3 Qingdao Institute of Bioenergy and Bioprocess Technology, Chinese Academy of Sciences, Qingdao 266101, China

**Keywords:** oil/water separation, crude oil absorption, chemical gradient, photothermal effect, wettability

## Abstract

Oil spills and oily wastewater discharges have posed severe threats to the ecosystem and human health, yet efficient cleanup and recovery remain huge challenges. The absorption of crude oil is especially difficult due to its high viscosity. In this study, we propose a strategy for the fast and highly selective absorption of crude oil as well as other oils and organic solvents with variable viscosity by combining the desert beetle’s back-inspired gradient hydrophobicity with the photothermal effect to enhance the absorption rate. The oil-absorbent material was prepared through the alkylsilane-based gradient chemical modification of MXene-polyurethane sponges. The hydrophobic gradient across the composite sponge offers an extra driving force for the selective oil wetting in the sponge. Owing to the synergistic effect between gradient wettability and photothermal heating, a faster absorption rate, in addition to the high separation rate, was achieved for a variety of oils, including thick crude oil, thin crude oil, and light diesel oil, compared to that without gradient wettability. The as-prepared material is robust with good repeatability for the oil absorption. The surface silane modification was also demonstrated to help prevent the oxidation of MXene, facilitating the long-term stability of the material. This study will enlighten the development of fast and highly selective liquid absorbents.

## 1. Introduction

In past decades, frequent oil spill accidents and the constant generation of oily wastewater have raised severe environmental, economic, and social concerns [1,2]. For instance, the Deepwater Horizon oil spill [3,4] in 2010, the pipeline oil leakage off the California coast [5] in 2021, and the Philippine oil tanker capsizing accident in 2024 have all resulted in long-term contamination of the local marine and ecological environment as well as huge economic damage. During these spills, the massive loss of both crude oil and fuel oil is also unfavorable for rational energy utilization, aggravating the fossil energy crisis to some extent [6]. On the other hand, oily wastewater, especially that from industrial and scientific activities, contains various kinds of toxic organic solvents that are a danger to the water system and human health [7]. Therefore, it is of great urgency and importance to develop new materials and methods for highly efficient oil cleanup as well as recovery.

Many methods have been utilized for oil spill cleanup, such as oil skimmers, absorbents, chemical dispersants, and combustion [8,9,10,11,12,13,14]. Among these methods, the most frequently used ones are absorbents and combustion, as they offer convenient and direct oil cleanup that reduces oil spread over time, benefiting instant damage control. However, the combustion treatment causes not only air pollution but also unnecessary loss of oil. The absorption method, on the other hand, offers the possibility for oil recovery in addition to cleanup, yet traditional absorbent materials, including cotton, wool fibers, and porous polymers, have low oil selectivity over water and lack recyclability. Inspired by the special wetting surfaces in nature, many superwetting materials have been designed to show high oil/water selectivity in separation-related applications [15]. During recent years, attention has been increasingly focused on the development of new porous absorbent materials with high capacity and repeatability, such as aerogels [16,17,18], hydrophobic polymer sponges [19,20,21], wood sponges [22,23], foams [24], and inorganic porous [25] materials. The low-surface-energy characteristic especially endows these absorbents with high hydrophobicity/lipophilicity, promising highly selective oil/water separation and oil recovery. However, the absorption and separation of crude oil remains difficult due to its high viscosity and low fluidity.

To address the global challenge of high-viscosity crude oil remediation, researchers developed the heat-assisted crude oil absorption method by utilizing porous materials with Joule heat or photothermal effect [26,27]. For example, Yu and collaborators [26] designed a novel graphene-coated polymer sponge to heat the crude oil by applying electricity, achieving an efficient decrease in oil viscosity and a significant reduction in absorption time. To overcome the reliance on external energy input for Joule heating, Hu’s team [28] developed a carbonized wood-based porous material, utilizing its photothermal effect to harvest sunlight for oil heating and cleanup. Since then, many materials, including carbon nanotubes [16], reduced graphene oxide [17], and carbon nanoparticles [22], have been integrated with porous matrices such as polymer sponges to fabricate photothermally enhanced oil absorbents. These materials also enable Joule heating under low-light conditions, thus allowing for 24/7 oil cleanup [29].

As the thermally enhanced absorbent materials provide new solutions for high-viscosity oil cleanup [30,31], it is of high necessity to further improve their oil absorption rate. The oil absorption rate is an essential factor during the actual oil spill cleanup, as the faster the cleanup, the lower the environmental pollution and economic losses caused by the oil diffusion will be. Previous oil absorption studies rely on the capillary effect generated by the hydrophobic porous network and the help of electric pumps [26,27,32]. If there are other driving forces to improve the absorption rate, we can then avoid the dependence on external equipment, achieving more convenient and economic oil cleanup, especially for offshore oil spills. However, this issue has not been considered and resolved until today.

Here, in this manuscript, we propose a strategy to improve the absorption rate of crude oil by combining gradient wettability and photothermal effect, for achieving both fast cleanup and highly selective recovery for oils with variable viscosity. This strategy is inspired by the water-harvesting desert beetle’s back, which features a patterned surface with alternating hydrophilic and hydrophobic regions [33,34]. Droplets on this surface can move from hydrophobic to hydrophilic zones driven by surface tension, facilitating efficient fog collection. Many materials with gradient wetting surfaces have been designed to induce the directional movement of liquid toward applications [35] such as heat transfer [36] and water collection [37], yet the exploration of oil absorption has not been carried out.

Building on this principle, this manuscript employs silane-based gradient chemical modification to construct a porous network with gradient hydrophobicity based on a polymer sponge. MXene nanosheets with excellent photothermal effect were also integrated into the porous network for the heating of crude oil to reduce its viscosity. The hydrophobic to superhydrophobic change from the bottom to the top of the modified sponge induces the surface-tension-driven transport of crude oil, accelerating its separation from water and enhancing the absorption speed while ensuring the high oil selectivity over water. The as-prepared materials exhibit a faster absorption rate of oils with variable viscosity, including thick crude oil, thin crude oil, and light diesel oil, shedding light on the next generation self-powered and highly selective oil absorption materials. Additionally, the excellent electrical conductivity of MXene also allows the composite sponge to utilize Joule heating, endowing the system with the possible 24/7 operational capability whenever needed.

## 2. Materials and Methods

Titanium aluminum carbide (Tl3AlC2, 200 mesh) was purchased from Science and Technology Company (Jilin, China). Lithium fluoride (AR, 99%) was obtained from Aladdin Biochemical Technology Company (Shanghai, China). Hydrochloric acid (HCl, AR) was purchased from Sinopharm Chemical Reagent Company (Shanghai, China). Liquid paraffin and trimethoxoctylsilane (97%) were received from damas-beta. n-decane (99%), n-dodecane (98%), and n-hexadecane (98%) were bought from McLean Biochemical Technology Company (Shanghai, China). Diesel fuel (API CF-4) and vegetable oils (blended oils) were purchased online at Taobao. Crude oil was received from Shengli Oilfield.

### 2.1. Preparation of the MXene Nanosheets

In total, 15 mL of concentrated HCl (12 M) was diluted with 5 mL of deionized (DI) water to obtain the 9 M HCl solution, and maintained at 39 °C in an oil bath. In total, 0.8 g of LiF was added slowly into the HCl solution within 10 min under stirring at 400 rpm. Then, 0.3 g of Ti_3_AlC_2_ MAX powder was slowly introduced into the above HCl/LiF solution, and the mixture was then allowed to react for 24 h at 37 °C. After that, the mixture was centrifuged at 4000 rpm for 5 min to discard the supernatant. The sediment was then washed repeatedly with DI water by cyclic centrifugations at 8000 rpm, until the pH of the supernatant exceeded 5. The sediment was then re-dispersed in 35 mL of DI water, sonicated in an ice bath for 13 min, and centrifuged at 4000 rpm for 20 min to obtain the supernatant with MXene nanosheets in it. The concentration of the MXene nanosheet suspension was measured by filtration and weighing, and set to 5 mg/mL for the following experiments.

### 2.2. The Coating of MXene Nanosheets on PU Sponge

The MXene nanosheets were coated on the PU sponge through a dip-coating process. Commercial PU sponge was cut into cube pieces with 8 cm^3^ in size. The PU sponge cubes were cleaned by sequential sonication in DI water and ethanol for 20 min, respectively, followed by drying at 60 °C for 2 h. The pre-cleaned sponge cubes were then immersed in the MXene nanosheet suspensions for 5 min, followed by vacuum drying at 60 °C for 20 min. This dip-coating step was repeated 4 times for full MXene nanosheet coverage. After DI water washing to remove the un-adsorbed nanosheets and vacuum drying at 60 °C for 1 h, the MXene-coated PU sponges, termed MXene@PU, were finally obtained.

### 2.3. Gradient Modification of Silane onto the MXene@PU

The MXene@PU was modified by silane through chemical vapor deposition (CVD). In order to realize the gradient silane modification, the as-obtained MXene@PU cubes were wrapped with plastic polyethylene film tightly to make sure that 5 sides of the cube were physically covered and only 1 side of it was exposed for the CVD. As a result, the silane vapor diffused into the sponge cubes only through the exposed side, leading to a silane molecule gradient across the sponge. In total, 50 µL of n-octyltrimethoxysilane (OTMS) was dripped onto the bottom of a reaction vessel, while the wrapped MXene@PU was mounted to the vessel lid, with its exposed side facing the silane liquid. The sealed reaction vessel was then heated at 30 °C for 75 min. After that, the OTMS modified MXene@PU, termed OTMS-MXene@PU, was placed in an oven at 60 °C for 6 h to eliminate the un-reacted OTMS residues. The OTMS-MXene@PU with gradient silane modification was therefore termed G-OTMS-MXene@PU. Meanwhile, silane modification without gradient treatment, i.e., without physical coverage during the modification, was also carried out. The corresponding samples were termed non-G-OTMS-MXene@PU.

### 2.4. Characterizations

The MXene nanosheets were characterized using transmission electron microscopy (TEM; JEM-F200, JEOL Ltd., Tokyo, Japan). The morphologies of PU sponge samples were analyzed by scanning electron microscopy (SEM; S-4800, Hitachi High-Technologies, Tokyo, Japan), and the chemical properties were analyzed by energy-dispersive X-ray spectroscopy (EDS) coupled with SEM, Fourier-transform infrared spectroscopy (FT-IR, Nicolet 6700, Thermo Scientific, Madison, WI, USA), and X-ray photoelectron spectroscopy (XPS; ESCALAB 250Xi, Thermo Fisher Scientific, East Grinstead, UK). Wettability of the materials was quantified using an optical tensiometer (Attension Theta Lite, Biolin Scientific, Espoo, Finland). To evaluate the photothermal performance, samples were illuminated with simulated sunlight from a 300 W Xenon lamp (CEL-HXF300, Beijing China Education Au-light Co., Ltd., Beijing, China), with light intensity calibrated to 1 sun (100 mW/cm^2^) or otherwise specified using an optical power meter (CEL-NP2000, CEAULIGHT, Beijing, China). Real-time thermal distribution was monitored by infrared thermography (FOTRIC 226s, FOTRIC Inc., Shanghai, China). The viscosity of the thin crude oil used was measured using a rheometer (HAAKE MARS 60, Thermo Fisher Scientific, Karlsruhe, Germany).

### 2.5. Characterization of the Absorption Properties

The experimental samples, i.e., the pristine PU, MXene@PU, and OTMS-MXene@PU cubes, 8 cm^3^ in size, were soaked with different kinds of oils for 5 min until absorption equilibrium, and then taken out and weighed using a balance (Quintix 35-1CN, Sartorius Lab Instruments GmbH & Co. KG, Göttingen, Germany). The absorption capacity ***Q*** was calculated by the equation as follows:

$$Q=\frac{{m_{2}-m}_{1}}{m_{1}}$$

where ***m***_1_ and ***m***_2_ are the initial mass and the mass after oil absorption of the samples, respectively. After that, the oil was fully squeezed out. The above procedure was repeated 5 times for a reliable and precise measurement.

When analyzing the selectivity of oil over water during the absorption, oil such as n-dodecane, n-hexadecane, or diesel fuel was added to 50 mL of DI water to prepare a mixed oil/water sample. The experimental samples, i.e., G-OTMS-MXene@PU and non-G-OTMS-MXene@PU cubes, with a size of 8 cm^3^, were placed into the mixture and absorbed until equilibrium was reached. The absorbed liquid mixtures were extruded using physical squeezing, and their purity was measured using an infrared oil meter (HCY50-1, WATENV, Shanghai, China).

During the testing process by the infrared oil meter, 0.2 mL of the tested sample and 100 mL of extraction solvent (tetrachloroethylene) were added into a separatory funnel and then shaken and extracted for 3 min. The extraction solvent was then measured to determine the oil concentration in it. The selectivity of oil over water was evaluated by calculating the oil separation rate (***η***) using the following equation:

$$\eta =\frac{C_{2}}{C_{1}}*100\%$$

where ***C*_2_** (mg/L) is the concentration of oil in the absorbed mixture, while ***C*****_1_** (mg/L) is the concentration of pure oil, all measured by the infrared oil meter.

### 2.6. Photothermal Heating Assisted Crude Oil Absorption

The experimental samples, i.e., the pristine PU, MXene@PU, and OTMS-MXene@PU cubes, 8 cm^3^ in size, were illuminated by the Xenon lamp with 100 mW cm^−2^ power density to simulate sunlight. The change in sample temperature was monitored by the infrared thermography in real time. After reaching the equilibrium of temperature, 0.15 g of the crude oil sample with high viscosity was then deposited onto the sample surface, while the absorption rate was monitored by optical recording using a cell phone camera.

### 2.7. Characterization of the Influence of Gradient Hydrophobicity on Absorption Rate

In total, 0.5 mL of liquid oil was added onto the surface of the experimental samples, i.e., the pristine PU, G-OTMS-MXene@PU, and non-G-OTMS-MXene@PU sponge cubes under ambient conditions (25 ± 1 °C). The absorption rate of oil by the corresponding sample was monitored in real time using an optical tensiometer equipped with a high-speed camera to calculate precisely the absorption time. The surface with low hydrophobicity of the G-OTMS-MXene@PU sponge cubes was placed facing upward for oil absorption.

### 2.8. Anti-Oxidation Test of the MXene with or Without Silane Modification

The experimental samples, i.e., the MXene@PU and non-G-OTMS-MXene@PU sponge cubes, were immersed in DI water for variable days (3 to 18 days) under ventilated and ambient conditions, after which the samples were taken out and dried in an oven at 60 °C for 6 h. The samples were then characterized by SEM, optical tensiometer, and infrared thermography to analyze their long-term stability.

## 3. Results

### 3.1. Preparation of the Silane-Modified Photothermal Sponge Absorbent

**Scheme 1:**
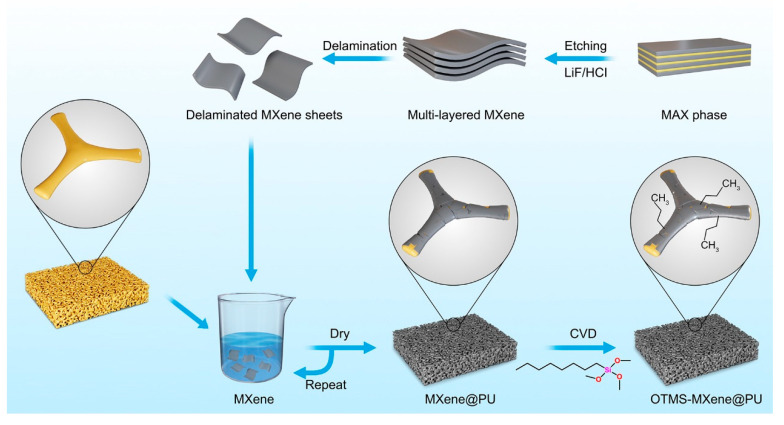
Schematic illustration of the preparation process of OTMS silane-modified MXene@PU absorbent.

The composite sponge absorbent was prepared by firstly coating with a layer of MXene nanosheets on the pristine polyurethane (PU) sponge and then gradient modification of octyltrimethoxysilane (OTMS) on the MXene surfaces (Scheme 1, see the Section 2.1, Section 2.2 and Section 2.3 for details). MXene nanosheets are used for providing the photothermal effect, while the gradient silane modification introduces gradient hydrophobicity into the composite sponge. MXene has been reported to have excellent photothermal effects and has been used in a variety of related applications [31]. Here, the Ti_3_C_2_T_x_ MXene nanosheets were prepared by the mild Al etching of the Ti_3_AlC_2_ MAX phase and the subsequent ultrasonic delamination process (Scheme 1). Tx represents the surface terminal groups generated during the etching, i.e., the -OH, C-O, and -F groups [38]. As shown from Figures S1 and S2, the as-prepared MXene nanosheets have a thickness of about 1 nm, after subtracting the gap between the nanosheet and the substrate during AFM measurements [39], and a lateral size of about 1.5 to 2 μm. The nanosheet suspension exhibited the Tyndall effect (Figure S3), indicating the good dispersion of nanosheets in water.

**Figure 1:**
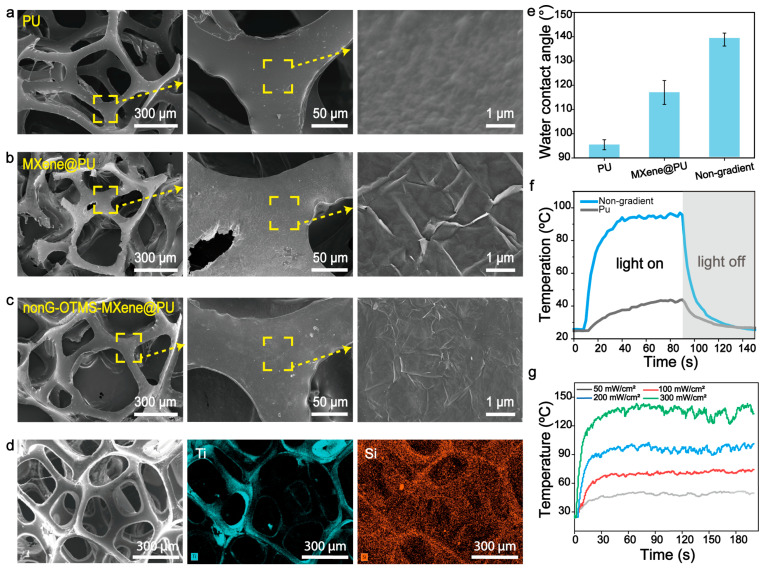
Preparation of the silane-modified photothermal composite sponge. (**a**–**c**) SEM images of the pristine PU (**a**), the MXene@PU (**b**), and the non-G-OTMS-MXene@PU (**c**). (**d**) EDS element analysis of the non-G-OTMS-MXene@PU, exhibiting the uniform distribution of Ti and Si in the sample. (**e**) Contact angle of the PU, MXene@PU, and non-G-OTMS-MXene@PU sponge samples. (**f**) Comparison of the photothermal effect of the pristine PU (black curve) and the non-G-OTMS-MXene@PU (blue curve) under light illumination of 200 mW cm^−2^. (**g**) Influence of the light intensity on the photothermal effect of OTMS-MXene@PU.

After the cyclic dip coating and drying process, MXene nanosheets were firmly adhered to the surface of the PU porous framework (termed MXene@PU, Scheme 1). SEM, as well as EDS characterizations (Figure 1a,b,d), indicate that the framework surface of PU sponge was fully and evenly covered by MXene nanosheets without influencing the porous structure. As shown from the FT-IR spectra (Figure S4), no new peaks appeared after loading the MXene nanosheets, suggesting that the MXene nanosheets adhered onto PU most probably due to the hydrogen bond and van der Waals forces between them rather than covalent bonds. Moreover, the -OH on the MhXene surfaces facilitates the modification of silane onto MXene@PU. Scheme 1 shows a typical process of the uniform OTMS silane modification onto MXene@PU by the CVD method. The uniformly OTMS-covered MXene@PU was then termed non-G-OTMS-MXene@PU, while non-G represents non-gradient modification, to differentiate from that of gradient silane modification described below.

As shown in Figure 1c, after OTMS modification, the surface of the MXene@PU turned smoother, suggesting the good coverage of OTMS molecules on MXene nanosheets. EDS analysis also indicates the well distribution of silane on the porous framework (Figure 1d). The FT-IR spectrum of OTMS-MXene@PU sponge showed a Si-O absorption peak at 808 cm^−1^, while the XPS spectrum and high-resolution Si2p spectrum also showed the existence of a Si-O bond, both demonstrating the successful modification of OTMS silane onto the MXene-PU (Figures S4 and S5).

To achieve selective oil absorption, the material itself should have good lipophilic and hydrophobic properties. The pristine PU sponge is biphobic (Figure 1e and Figure S6). After MXene and silane attachment, it showed that the hydrophobicity of the PU sponge was gradually increased, from 95° for the pristine PU, to 120° for MXene@PU, to 140° for the non-G-OTMS-MXene@PU (Figure 1e), while the lipophilicity was also enhanced from 84° for the pristine PU and 0° for the non-G-OTMS-MXene@PU (Figure S6), therefore endowing the OTMS-MXene@PU with the ability to absorb oil highly selectively.

The photothermal effect of OTMS-MXene@PU was also verified by monitoring the temperature change in the sponge samples under simulated white light illumination. As shown in Figure 1f and Figure S7, the pristine PU sponge exhibited negligible photothermal effect, with the temperature rising from 27 °C to 40 °C under light illumination (200 mW cm^−2^). In contrast, the temperature of non-G-OTMS-MXene@PU increased to more than 90 °C within 40 s and reached the platform, and gradually decreased to room temperature after the light was off. Moreover, as the light intensity increased from 50 to 300 mW cm^−2^ (0.5 to 3 suns), the temperature of non-G-OTMS-MXene@PU also increased correspondingly, with the stable temperature reaching over 130 °C under 300 mW cm^−2^ light illumination (Figure 1g).

### 3.2. Gradient Hydrophobicity Enhanced Oil Absorption Rate

**Figure 2:**
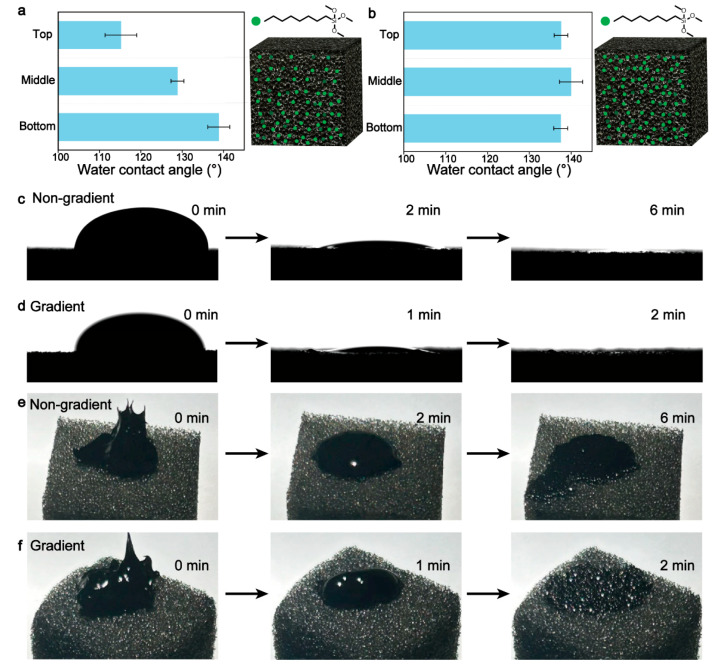
Gradient hydrophobicity enhanced oil absorption rate. (**a**) Gradient hydrophobicity of the G-OTMS-MXene@PU. (**b**) Uniform hydrophobicity of the non-G-OTMS-MXene@PU. The green dots in the images represent n-octylsilane molecules (**c**,**d**) Comparison of the absorption rate of the thin crude oil samples between the non-G-OTMS-MXene@PU and the G-OTMS-MXene@PU sponge cubes. (**e**,**f**) Comparison of the absorption rate of the thick crude oil samples between the non-G-OTMS-MXene@PU and the G-OTMS-MXene@PU sponge cubes under 1 sun illumination.

The above results confirmed the reliability of OTMS silane modification in improving the hydrophobicity of PU sponge and the excellent photothermal effect introduced by MXene functionalization. Based on the non-gradient silane modification parameters, we further carried out the gradient silane modification. By physical blockage of the five sides of MXene-PU sponge cubes, the silane vapor could only penetrate the porous framework through one direction (see the Section 2.3 for details), therefore resulting in the gradient silane modification (termed G-OTMS-MXene@PU). As shown in Figure 2a,b, the contact angles of the G-OTMS-MXene@PU from the top to the bottom were 115° ± 4°, 129° ± 2°, and 139° ± 3° (Figure 2a), respectively, while the contact angles of the non-G-OTMS-MXene@PU were kept at about 139° ± 3° throughout the sponge sample (Figure 2b), strongly demonstrating the successfully gradient modification of the OTMS silane and the construction of gradient hydrophobicity. Moreover, it was verified that the photothermal effect of the G-OTMS-MXene@PU was kept the same as that of the non-G-OTMS-MXene@PU (Figure S8).

We then systematically investigated the influence of gradient hydrophobicity on the absorption rate of different kinds of oils. In order to rule out the influence of temperature change on absorption rate during the experiments, crude oil mixed with liquid paraffin, which is a common crude oil type [40], was first used for the test. The thin paraffinic crude oil has a certain degree of fluidity at room temperature compared to the thick crude oil. Nevertheless, the viscosity of this thin crude oil mixture, which was measured to be about 1857.4 mPa·s at 25 °C and 91.7 mPa·s at 40 °C (Figure S10), is still far higher than that of the commonly used oils such as diesel fuel with a reported viscosity of 2.0–4.5 mPa·s at 40 °C [41]. In total, 0.5 mL of the thin crude oil mixture was added to the pristine PU, the uniformly modified non-G-OTMS-MXene@PU, and the gradient-modified G-OTMS-MXene@PU, respectively, while the side with the low hydrophobicity of the G-OTMS-MXene@PU was moved upward to contact the oil. The size of these three sponge cubes was kept the same, i.e., 8 cm^3^. The results show that the pristine PU has poor absorption ability for the thin crude oil sample (Figure S9). There was still about half of the oil left on the sponge surface after 12 min. In contrast, sponge samples with uniform silane modification (non-G-OTMS-MXene@PU) showed better absorption capability owing to their higher hydrophobicity compared to the pristine PU (Figure 2c). However, the gradient-modified G-OTMS-MXene@PU fully absorbed the same amount of oil within 2 min (Figure 2d) and exhibited a significantly higher crude oil absorption rate compared to the non-G-OTMS-MXene@PU (6 min for a total absorption), indicating that the gradient hydrophobicity generated by gradient silane modification can indeed promote the oil absorption speed. In addition, we also tested the difference in absorption rate for light diesel fuel. The results again demonstrate that the gradient-modified sponge absorbed the same amount of oil significantly faster than the uniformly modified ones, indicating a higher absorption rate (Figure S11).

Moreover, we tested the absorption of thick crude oil with bare fluidity with the help of photothermal heating, and found the same result (Figure 2e,f). First, we verified the influence of temperature on the viscosity of crude oil using the thin crude oil sample mixed with liquid paraffin, as it is difficult to directly measure the viscosity of the 100% thick crude oil sample. The result showed that the viscosity of crude oil reduced quickly with the increase in its temperature and reached nearly the minimum when the temperature was around 40 °C (Figure S10). Such low viscosity after heating allows the crude oil sample to be absorbed much more quickly by the OTMS-MXene@PU compared to that at room temperature (Figure S12). These results indicate the feasibility of using the G-OTMS-MXene@PU to heat the crude oil when referring to the photothermal capability of the absorbents, as shown in Figure 1f,g. Subsequently, thick crude oil samples with bare fluidity of the same mass, 0.15 g, were added onto the non-G-OTMS-MXene@PU and G-OTMS-MXene@PU, respectively, under 100 mW cm^−2^ light illumination. The pristine PU was also tested for comparison. The results show that the crude oil sample could not be absorbed by the pristine PU due to the lack of photothermal effect of the PU (Figure S13). On the contrary, the crude oil was totally absorbed by the G-OTMS-MXene@PU in just 2 min, while the crude oil sample with the same mass was fully absorbed by the non-G-OTMS-MXene@PU in 6 min—much longer than that of the gradient one (Figure 2e,f).

The above results strongly demonstrate, for the first time, the advantage of the gradient hydrophobicity in enhancing the oil absorption rate, not only for high-viscosity thick crude oil but also for low-viscosity light oils. Nevertheless, it is worth noting that the linear hydrophobic gradient generated here might not be very convenient for usage during the real application. It was shown that, by using the other five sides of the G-OTMS-MXene@PU sponge cubes to contact with the oil, the oil absorption rate was comparatively lower than that using the low hydrophobic side (Figure S11 compared to Figure S14) due to the weak hydrophobic gradient from these sides. A radical gradient from the center of the sponge cubes will be favored during a real oil spill scenario and is worth exploring in further studies.

### 3.3. Versatility and Repeatability of the G-OTMS-MXene@PU Sponges for Highly Selective Oil Absorption

**Figure 3:**
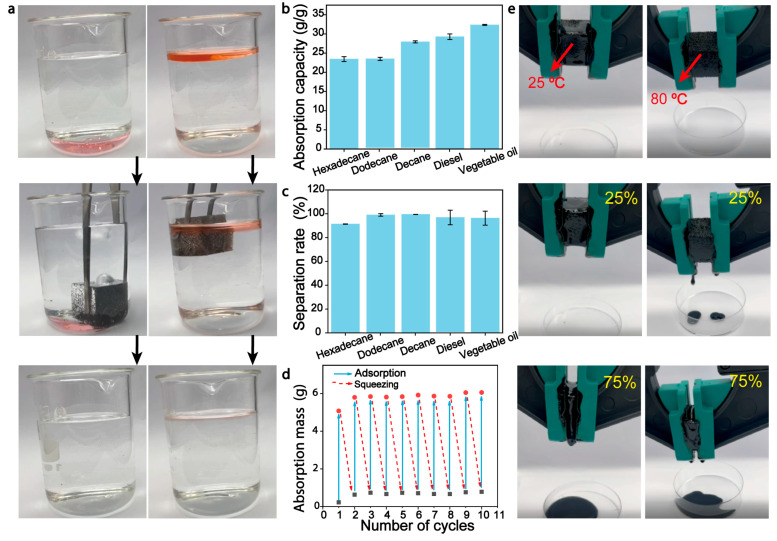
Versatility and repeatability of the G-OTMS-MXene@PU sponges for highly selective oil absorption. (**a**) The absorption of heavy oil and light oil (both dyed for better view) from the oil/water mixtures. (**b**,**c**) The absorption capacity (**b**) and separation rate (**c**) of the G-OTMS-MXene@PU for different oil types. (**d**) The stability of the absorption capacity during 10 cycles of the absorbing–squeezing process. (**e**) Mechanical extrusion of the absorbed crude oil under different temperatures.

Experiments show that the as-prepared sponge samples are both porous and superhydrophobic. The porous structure should ensure a large surface area and a high absorption capability, while the high hydrophobicity should endow a high oil selectivity over water during the absorption. To further verify the oil-absorbing capability of the G-OTMS-MXene@PU sponges, we utilized a variety of oils and organic solvents for the corresponding tests. For convenience, oils and organic solvents with low viscosity were used. As shown in Figure 3a, the G-OTMS-MXene@PU sponges can efficiently absorb most of the oil in a matter of seconds during a one-time absorption process from the oil/water mixtures, for both the heavy ones and the light ones, compared to the density of water. The residual oil should be attributed to the absorption saturation of the sponge sample. It can be expected that by using multiple absorption cycles, the oils could be removed as cleanly as possible.

The corresponding oil absorption capacity and separation rate of the G-OTMS-MXene@PU were then measured by soaking the sponge samples in a variety of oils and organic solvents and then squeezing the liquid mixture out for weighing and IR analysis. Due to its hydrophobic (with a contact angle of 95 ± 3°, Figure 1e) and oleophobic (with a contact angle of 85 ± 3°, Figure S6) nature, the pristine PU exhibited poor oil absorption and separation capability (Figure S15). In contrast, as shown in Figure 3b, the G-OTMS-MXene@PU can absorb oils and organic solvents 20 ~ 30 times its own weight, exhibiting a relatively high absorbing capacity. The differences in the absorbing capacity between the oils and solvents should be attributed to the differences in their viscosity. The oil separation rate is also high, which is around 90% for these different kinds of oils and organic solvents. In addition, the absorption capacity and separation rate are also similar to those of the non-G-OTMS-MXene@PU (Figures S16 and S17), confirming that the gradient-modified sponge absorbent does not compromise itself on these capabilities.

Moreover, the G-OTMS-MXene@PU also showed high recyclability in addition to the above versatility. As shown in Figure 3d, the absorbing capacity remained relatively stable during the 10 absorbing–squeezing cycles tested with decane as the model oil. With the help of the photothermal effect of the sponges, the crude oil could be absorbed and also be squeezed out easily for recovery after the absorption (Figure 3e). When the deformation of the G-OTMS-MXene@PU sponge samples reached 25%, the absorbed crude oil at 80 °C was already squeezed out, while the crude oil at room temperature just started to drip down when the deformation reached 45%. More crude oil can be squeezed out under high temperature when the sponge deformation is 75%. The oil recovery ratio was about 90% with high stability during the cyclic absorbing–squeezing process (Figure S18).

### 3.4. Anti-Aging Property of the Silane-Modified MXene

The aging of MXene nanosheets is a general concern during MXene-related studies and applications. There have been continuous efforts in exploring the anti-oxidation and anti-degradation methods for MXene, including surface coating, surface modification, and storage in solvents [42]. During our studies, we noticed that the silane-modified OTMS on the MXene nanosheets seemed to form a dense and complete molecular layer on the MXene surfaces (comparing Figure 1b,c), indicating that the silane modification should have an effect to a certain degree in protecting the MXene nanosheets from aging. It is widely recognized that water with dissolved oxygen is a critical factor that causes the oxidation and aging of MXene materials [43], while there have been reports to show that non-polar oils do not react with MXene chemically, and some high-viscosity oils can even form a protective layer on the MXene surface [44]. As water is usually the majority component during the oil absorption from oil/water mixtures, and the types and percentages of oils vary widely, we chose water immersion as the evaluation method to study the influence of water on the aging of the as-prepared sponge samples. Dissolved oxygen also existed in the water during the experiments as the system was put in a ventilated condition.

**Figure 4:**
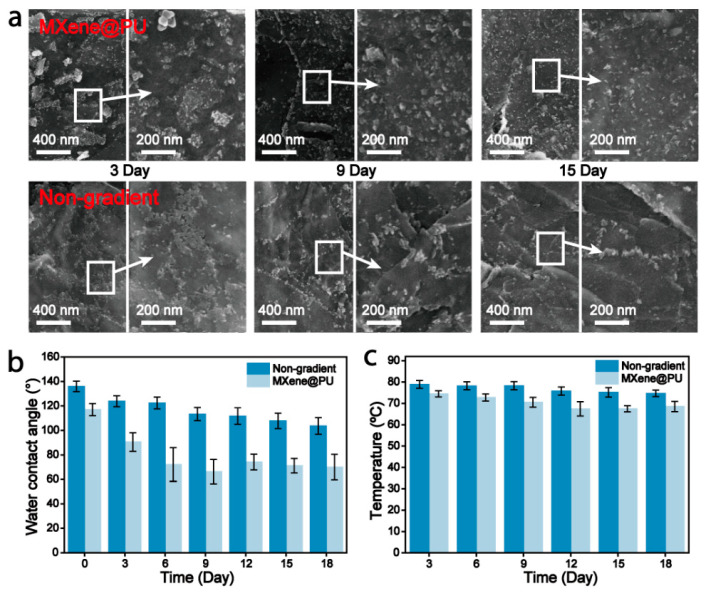
Anti-aging effect of the silane modification to MXene and the related influence on the hydrophobicity of sponge absorbents. (**a**) Influence of water immersion time on the surface microstructures of MXene nanosheets on the PU sponge framework. The upper line of the SEM images represents the un-modified MXene-PU, while the lower line of the SEM images represents the uniformly modified non-G-OTMS-MXene@PU. (**b**) Influence of water immersion time on the water contact angle on the surfaces of un-modified MXene-PU and non-G-OTMS-MXene@PU. (**c**) Influence of immersion time on the photothermal effect of the modified and un-modified sponge samples.

In detail, the un-modified MXene-PU and the OTMS silane-modified MXene@PU (non-G-OTMS-MXene@PU) sponge samples were divided into groups and soaked in water for various amounts of days under ventilated and ambient conditions, after which the sponge samples were taken out, squeezed, and dried for the following analysis. SEM characterizations show that, during the 18 days of tests, the silane-modified MXene-PU (non-G-OTMS-MXene@PU) indeed exhibited slower and minor aging traits, with fewer fragments shown only on the edges of the nanosheets (Figure 4a). The surfaces of the silane-modified nanosheets were still smooth even after 15 days of immersion. On the contrary, the oxidation degree of the un-modified nanosheets was quicker and more severe (Figure 4a), with more fragments shown on both the surfaces and edges of nanosheets starting significantly from the beginning days.

In addition, the corresponding hydrophobicity of the un-modified and modified sponge samples after varying days of immersion was also evaluated, as the hydrophobicity plays a key role in oil absorption. The results show that after the silane modification, the water contact angle on the sponge surface reduced more slowly compared to that without modification (Figure 4b). After 18 days of immersion, the water contact angle of MXene@PU reduced to less than 70°, while the contact angle of non-G-OTMS-MXene@PU was still maintained at more than 100°. The results demonstrate that the saline modification can indeed slow down the degradation of MXene, therefore maintaining the hydrophobicity of the sponge absorbent as long as possible. Although the influence of silane modification on the long-term photothermal effect is not that significant (Figure 4c), the above results confirm the anti-aging effect of the silane modification to MXene and strengthen the importance of silane modification for ensuring the high hydrophobicity and oil separation performance of MXene-based photothermal sponge absorbents.

## 4. Discussion

Inspired by the efficient water-harvesting mechanism of the desert beetle’s back, this work innovatively designed a biomimetic gradient wettability photothermal porous network for oil cleanup and recovery. By integrating the advantages of superwetting oil/water separation and thermally enhanced oil absorption, the as-prepared composite sponge absorbent achieves rapid separation and recovery of a variety of oils and organic solvents with varying viscosity, with a high oil separation rate and recyclability. The gradient hydrophobicity especially enhances the absorption rate of oils such as crude oil and diesel oil, which is especially favorable for instant and fast clean-up of oils during offshore oil leakage accidents. Moreover, the study also provides an inspiration for the application of gradient wettability on advanced surface/interface materials. In the following studies, efforts will be especially focused on the optimization of gradient wettability, for example, to obtain the change from hydrophobicity to super-hydrophobicity (>150°), to achieve faster oil absorption and higher oil selectivity in water. The scalability of the system is also worth exploring to improve its practical applications.

## Data Availability

The original contributions presented in this study are included in the article/Supplementary Materials. Further inquiries can be directed to the corresponding author.

## Supplementary Materials

The following supporting information can be downloaded at: https://www.mdpi.com/article/10.3390/biomimetics10090585/s1; Supplementary information and discussions on material characterization of MXene nanosheets and the composite sponge absorbent, the nanosheet morphology, G-OTMS-MXene@PU bonding form, hydrophobic and lipophilic characteristics, photothermal effect, and absorption capacity of G-OTMS-MXene@PU (Figures S1–S18). This material is available free of charge.

## Abbreviations

The following abbreviations are used in this manuscript:

PU	Polyurethane
OTMS	Octyltrimethoxysilane
DI	Deionized
CVD	Chemical vapor deposition
