# Supplementary material for: Photothermal Porous Material with Gradient Hydrophobicity for Fast and Highly Selective Oil/Water Separation and Crude Oil Recovery
Source: Biomimetics (Basel). 2025 Sep 3;10(9):585. doi: 10.3390/biomimetics10090585 (PMC12467646; doi:10.3390/biomimetics10090585)
Supplement: Supplementary file 1 [file biomimetics-10-00585-s001.zip › biomimetics-3834783-supplementary.pdf]

Supporting Information for

Photothermal porous material with gradient hydrophobicity for enhanced crude oil absorption rate toward fast and highly selective oil/water separation and oil recovery

Tianwen Wang[1,2], Song Song[1,2], Shiwen Bao[1,2], Yanfeng Gong[1,2], Yujue Wang[1,2], Chuncai Wang[1,2], Wenshao Ma[1,2], Nuo Liu[1], Kunyan Sui[1], Jun Gao[2,3] and Xueli Liu[1*]*

[1]Key Laboratory of Marine Bio-based Fibers of Shandong Province, College of Materials Science and Engineering, Key Laboratory of Shandong Provincial Universities for Advanced Fibers and Composites, Qingdao University, Qingdao 266071, P. R. China

[2]Qingdao Institute of Bioenergy and Bioprocess Technology Chinese Academy of Sciences, Qingdao 266101, P. R. China

[3]Shandong Energy Institute, Qingdao 266101, P. R. China

*Corresponding author

E-mail address: jun.gao@qibebt.ac.cn (J. Gao); liuxl@qdu.edu.cn (X. Liu).

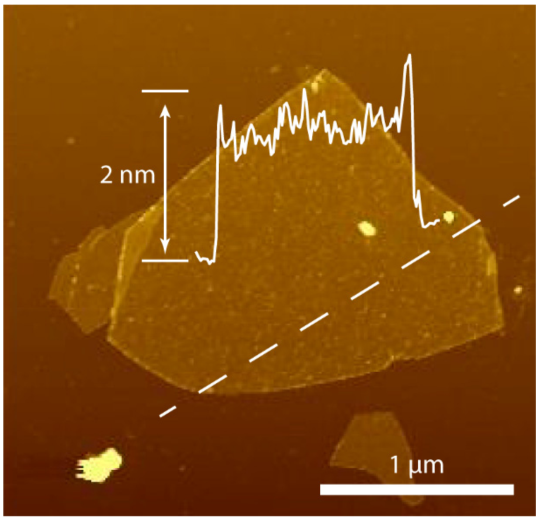

Figure S1. AFM image of the MXene nanosheets.

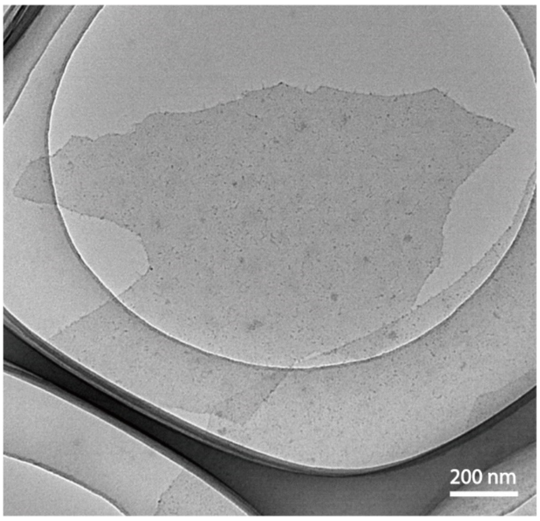

Figure S2. TEM image of the MXene nanosheets.

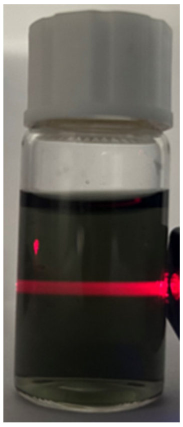

Figure S3. Tyndall effect of the MXene nanosheet dispersion.

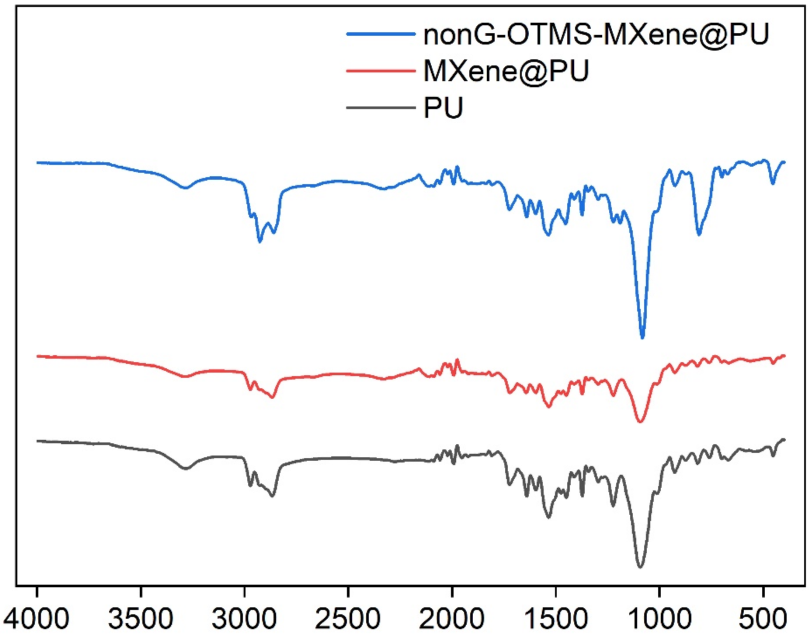

Figure S4. FT-IR spectra of the non-G-OTMS-MXene@PU, MXene@PU, and PU sponges.

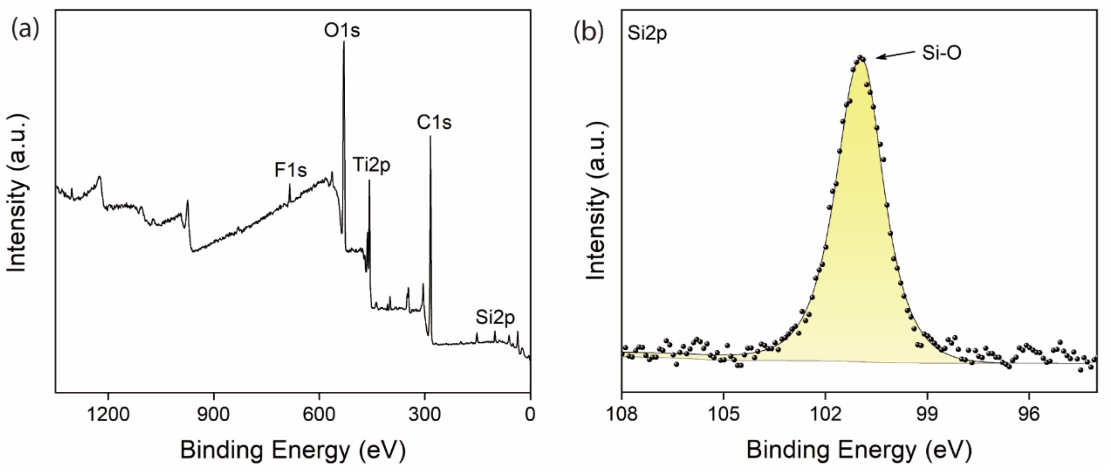

Figure S5. XPS spectrum (a) and the high-resolution Si_{2p} spectrum (b) of non-G-OTMS-MXene@PU.

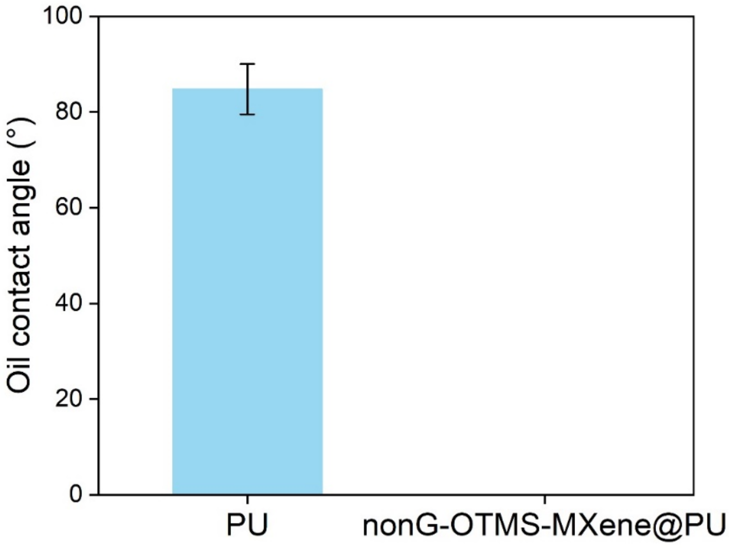

Figure S6. The contact angle of oil on the pristine PU and the non-G-OTMS-MXene@PU.

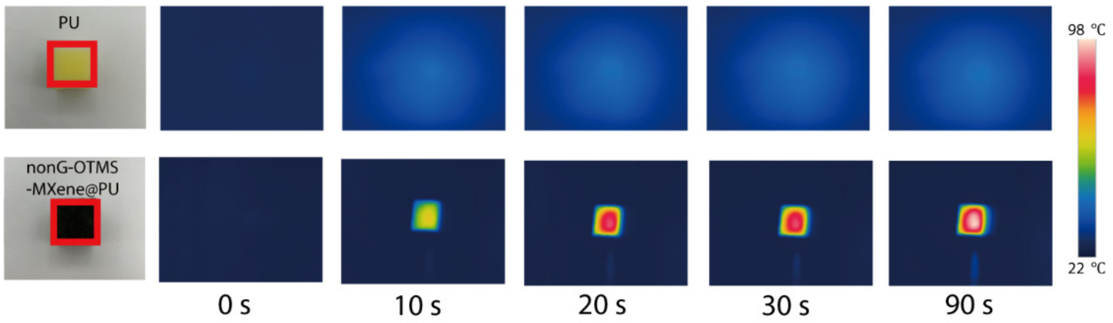

Figure S7. The change in temprature of the prinstine PU and the non-G-OTMS-MXene@PU sponge cubes under light illumination with a power density of 200 mW cm^{-2}.

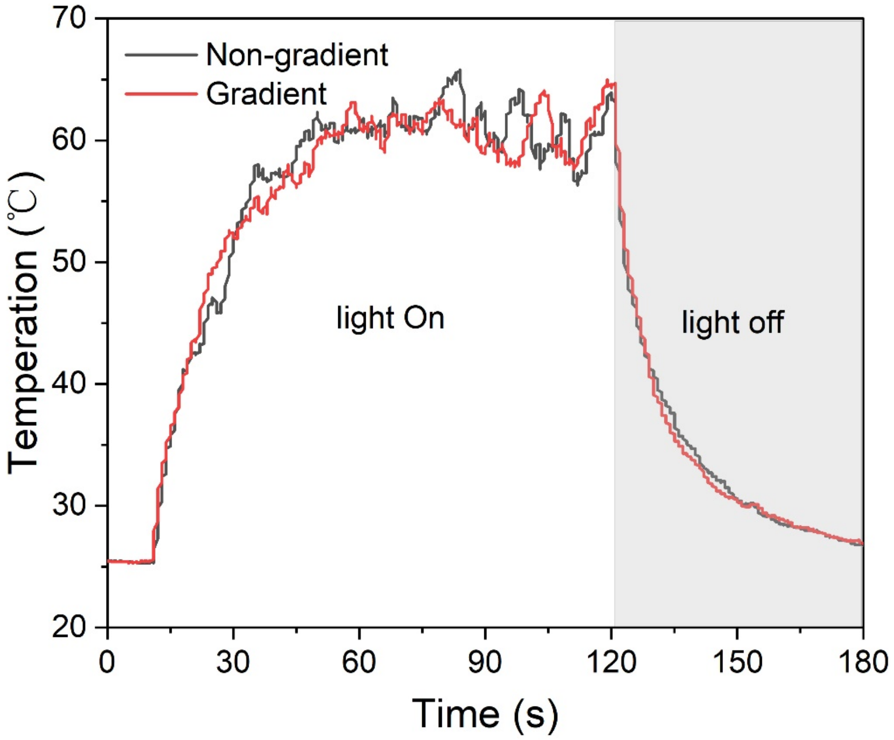

Figure S8. Comparation of the photothermal effect between the G-OTMS-MXene@PU and non-G-OTMS-MXene@PU sponge samples under 1 sun illumination (100 mW cm^{-2} while light).

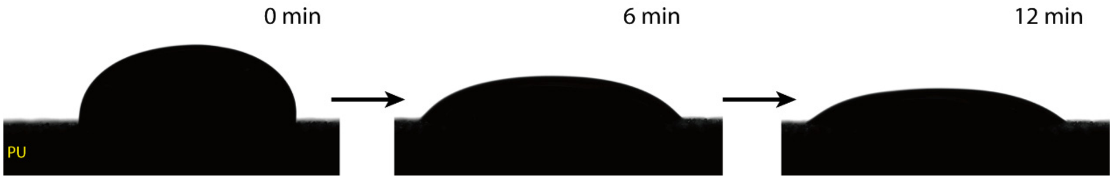

Figure S9. The absorption of thin crude oil along with time by the pristine PU sponge samples.

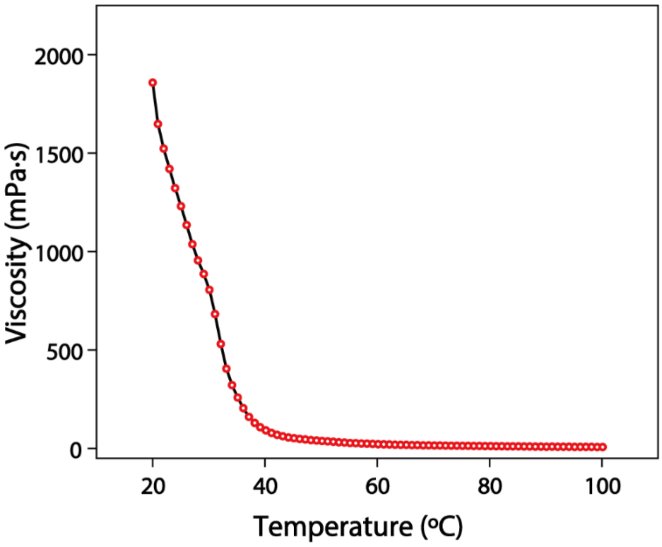

Figure S10. The change in viscosity of the thin crude oil sample mixed with liquid paraffin along with the temperature.

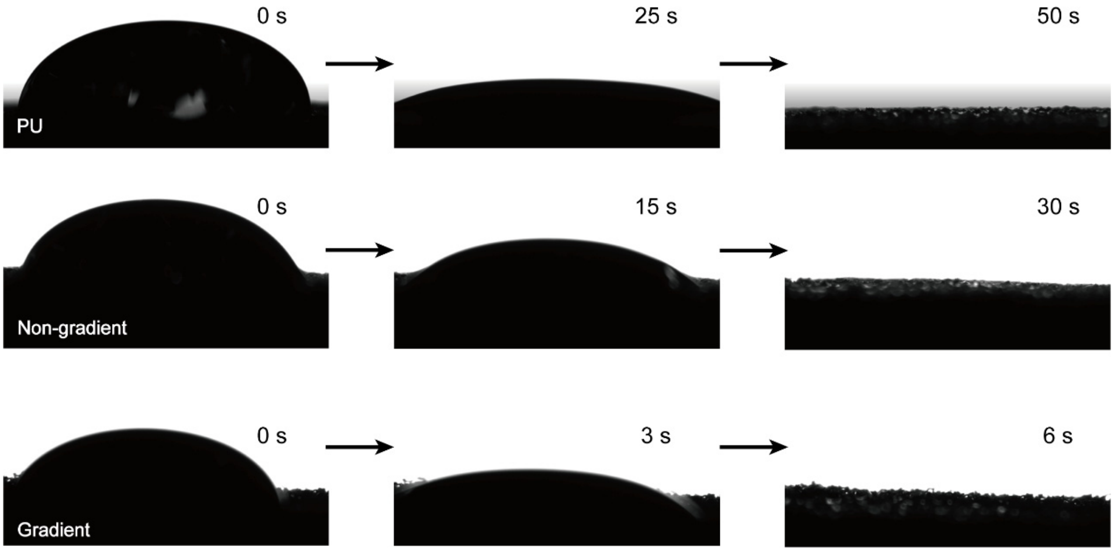

Figure S11. Comparison of the absorption rate of diesel fuel between the pristine PU, the non-G-OTMS-MXene@PU, and the G-OTMS-MXene@PU sponge samples.

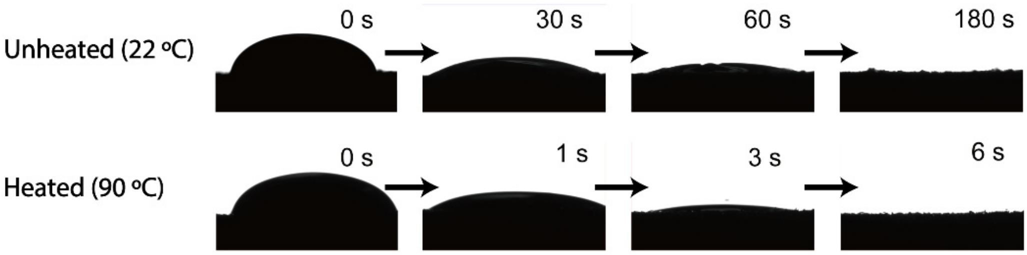

Figure S12. Comparison of the absorption rate of the thin crude oil samples under different temperature by the G-OTMS-MXene@PU sponge.

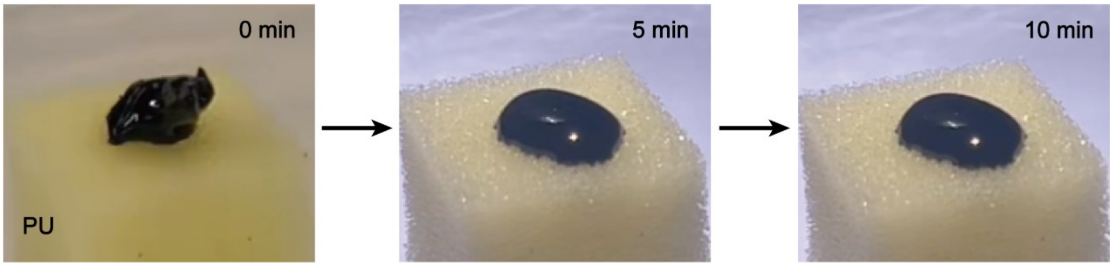

Figure S13. The crude oil absorption ability of the pristine PU sponge under light illumination (1 sun). The crude oil could not be absorbed by the PU sponge due to the lack of photothermal effect of pristine PU. The crude oil was slightly softened only due to its own light absorption.

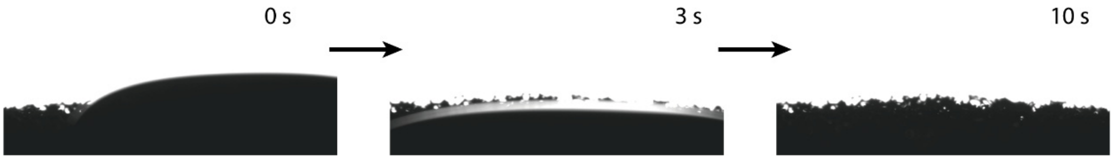

Figure S14. The absorption of diesel oil by using the other fives sides of the G-OTMS-MXene@PU to contact with the oil. The results show that it took longer time by using the five sides for contact (about 10 s) to fully absorb the same amount of oil compared to that using the low hydrophobic side (about 6 s, Figure S11).

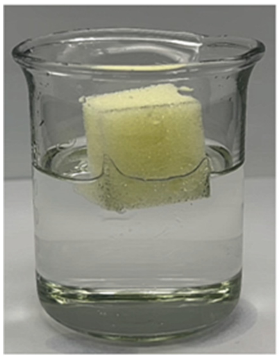

Figure S15. Oil absorption of the pristine PU. Due to the hydrophobic (with a contact angle of 95±3°, Figure 1e) and oleophobic (with a contact angle of 85±3°, Figure S6) nature of the pristine PU, the oils such as n-dodecane cannot be absorbed by the PU sponge spontaneously.

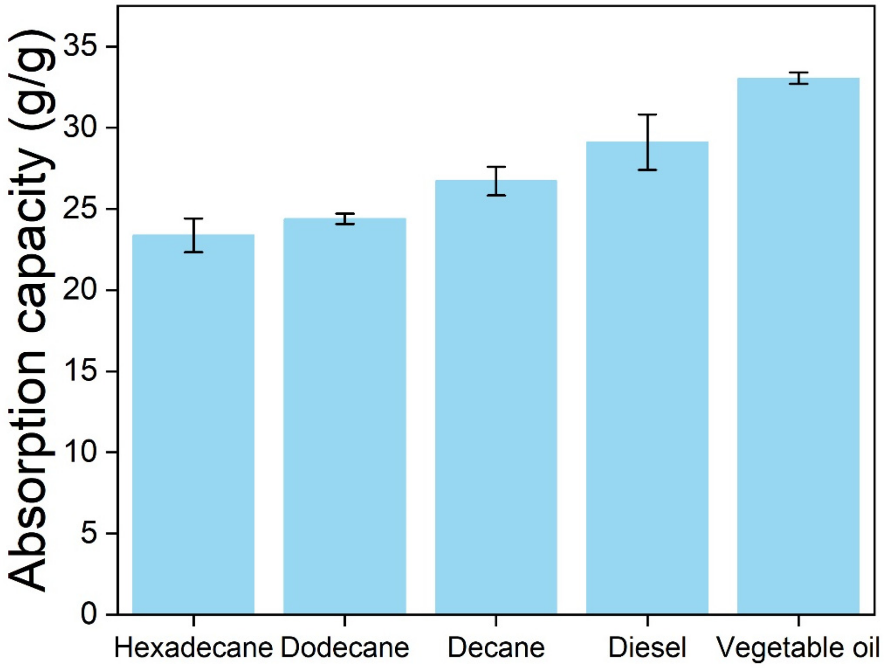

Figure S16. The absorption capacity of the non-G-OTMS-MXene@PU for different oil types.

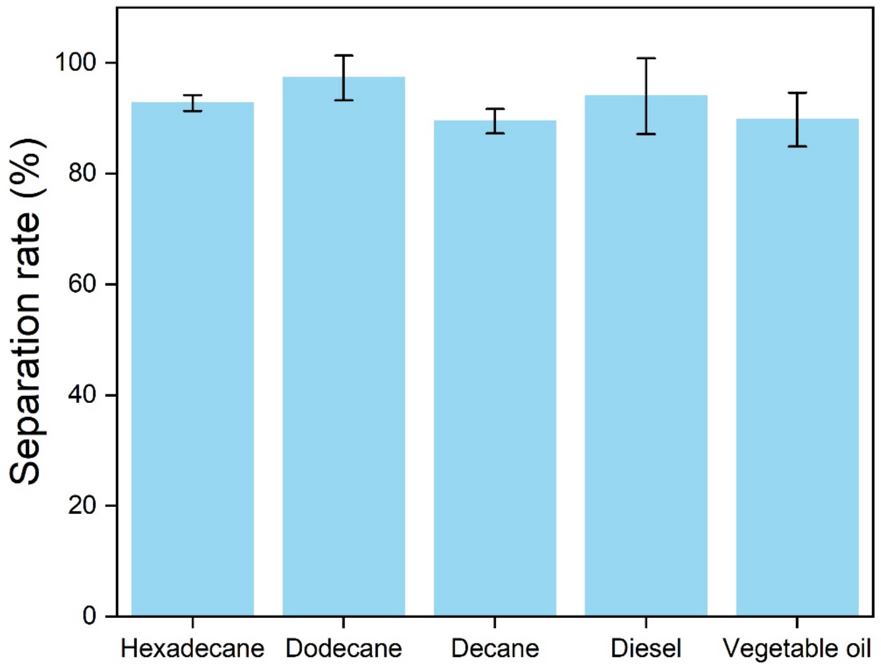

Figure S17. The absorption selectivity of the non-G-OTMS-MXene@PU for different oil types.

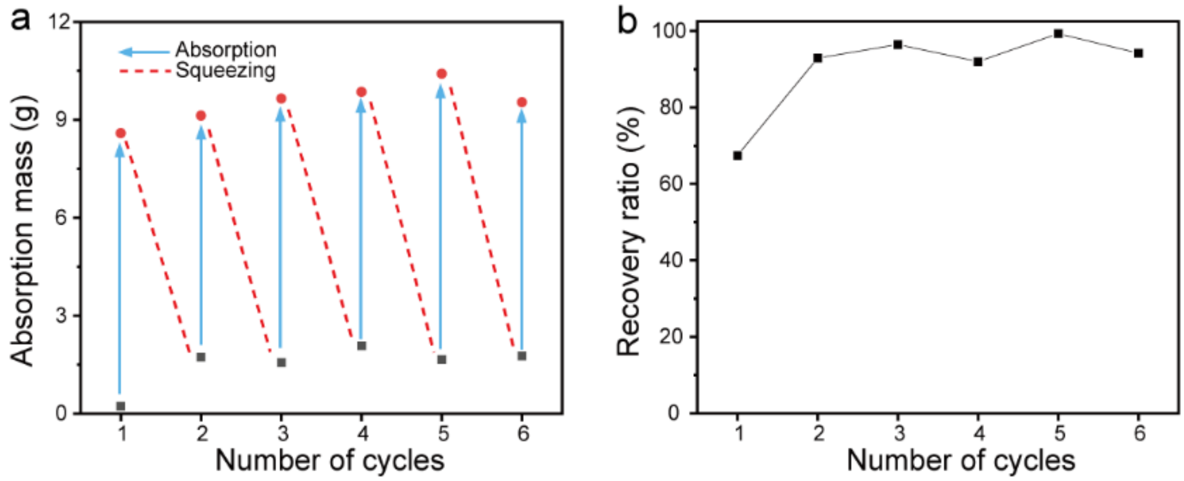

Figure S18. Cyclic absorption and recovery of the crude oil.
